# Gene-Wide Analysis of Aquaporin Gene Family in *Malus domestica* and Heterologous Expression of the Gene *MpPIP2;1* Confers Drought and Salinity Tolerance in *Arabidposis thaliana*

**DOI:** 10.3390/ijms20153710

**Published:** 2019-07-29

**Authors:** Haili Liu, Leilei Yang, Miaomiao Xin, Fengwang Ma, Jingying Liu

**Affiliations:** 1 State Key Laboratory of Crop Stress Biology for Arid Areas/Shaanxi Key Laboratory of Apple, College of Life Sciences, Northwest A&F University, Yangling 712100, China; 2 State Key Laboratory of Crop Stress Biology for Arid Areas/Shaanxi Key Laboratory of Apple, College of Horticulture, Northwest A&F University, Yangling 712100, China

**Keywords:** apple, aquaporin, functional analysis, stress tolerance

## Abstract

The aquaporins (AQPs) are a family of integral membrane proteins involved in the transcellular membrane transport of water and other small molecules. A scan of the apple (*Malus domestica*) genome revealed the presence of 42 genes encoding putative AQPs. Based on a phylogenetic analysis of the deduced peptide sequences of the AQPs generated by *Arabidopsis thaliana*, poplar (*Populus trichocarpa*), and rubber (*Hevea brasiliensis*), the apple AQPs were each assigned membership of the five established AQP subfamilies, namely the PIPs (eleven members), the TIPs (thirteen members), the NIPs (eleven members), the SIPs (five members), and the XIPs (two members). The apple AQPs included asparagine-proline-alanine (NPA) motifs, an aromatic/arginine (ar/R) selectivity filter, and the Froger’s positions. The heterologous expression of *MpPIP2;1* in *A. thaliana* was shown to enhance the level of tolerance exhibited against both drought and salinity.

## 1. Introduction

The aquaporins (AQPs) represent a family of integral membrane proteins, and form channels which allow the transport of water and other small molecules across membranes [1]. These proteins are produced by species across the phylogenetic spectrum, from microbes to plants and animals [2]. A typical aquaporin feature six transmembrane (TM1–TM6) helices (H1–H6) and five connecting loops (LA–LE); both their carboxylic and amino terminals lie on the cytoplasmic side, while two half helices formed the seventh TM helix by the opposite LB and LE dipping into the membrane. Given their general conservation across many AQPs, the asparagines-proline-alanine (NPA) motifs, the aromatic/Arginine (ar/R) selectivity filter formed by four residues (F58-H182-C191-R197 in AQP1) [3], and Froger’s positions (P1–P5 residues, T116-S196-A200-F212-W213 in AQP1) [4], are considered to be important for function. AQPs are tetrameric proteins and each monomer is functional independently as a channel. Furthermore, the fifth channel, which forms through the middle of tetramer array, has been suggested to conduct gases, like CO_2_ [5].

The survival and growth of a plant depends on its ability to maintain a sufficient level of tissue hydration. Proteins referred to as AQPs are known to represent an important component of the regulatory machinery used by plants for this purpose. AQPs have been shown to exert control over germination, since pea seeds imbibed in the presence of the AQP inhibitor mercury do not germinate [6]. The correlation established between the elongation of the *Ricinus communis* seedling hypocotyl and the abundance of the AQP-encoding gene *PIP2-1* has been taken to imply that AQPs also have a role in seedling growth [7]. The product of the tobacco gene *NtAQP1* has been shown to facilitate CO_2_ membrane transport, and to contribute both to photosynthesis and stomatal movement [8,9]. The products of the strawberry AQP-encoding genes *FaPIP1;1* and *FaNIP1;1* both appear to be involved in the transport of water into the fruit [10,11]. A number of authors have reported that plant AQPs respond to external stress, triggering physiological adjustments which act to maintain the plant’s hydration status [12,13,14,15,16].

Plant genomes encode a substantial number of AQPs: There are 35 such genes in the *Arabidopsis thaliana* genome [17], 72 in the soybean (*Glycine max*) genome [18], and 55 in the poplar (*Populus trichocarpa*) genome [19]. Based on their peptide sequences, higher plant AQPs have been classified into five subfamilies, namely the plasma membrane intrinsic proteins (PIPs), the tonoplast intrinsic proteins (TIPs), the Nod26-like intrinsic proteins (NIPs), the small and basic intrinsic proteins (SIPs), and the uncharacterized intrinsic proteins (XIPs). PIPs, TIPs, NIPs, and SIPs have been found in most higher plants, while to date XIPs have not been identified in *Brassicaceae* and monocots [20,21]. The most abundant of the AQPs are the PIPs and TIPs, most of which are associated with, respectively, the plasma membrane and the vacuolar membrane. Here, the family of apple (*Malus domestica*) AQPs has been characterized at the phylogenetic level, at the level of the chromosomal distribution of their encoding genes and with respect to the content of their functional domains. The effect on drought and salinity tolerance of one of these genes was explored by heterologously expressing it in *A. thaliana*.

## 2. Results

### 2.1. The Family of AQPs Encoded by the Apple Genome

A set of 67 candidate apple AQPs were identified by a key word search of the NCBI protein database. Several of these were discarded on the basis that they were either likely duplicates or represented a truncated sequence. The sequences of previously identified apple AQPs were used as queries of the whole apple genome sequence to identify additional members. The outcome was the identification of 42 putativeAQPs (Table 1). The range in length of their products was 236–309 residues and in their molecular weight was 25.1–33.2 KDa. The pI (isoelectric point) value of the presumptive AQPs varied from 4.86 to 9.97. Twenty nine of the forty two apple AQPs included six transmembrane domains.

### 2.2. Phylogenetic Analysis

**Figure 1:**
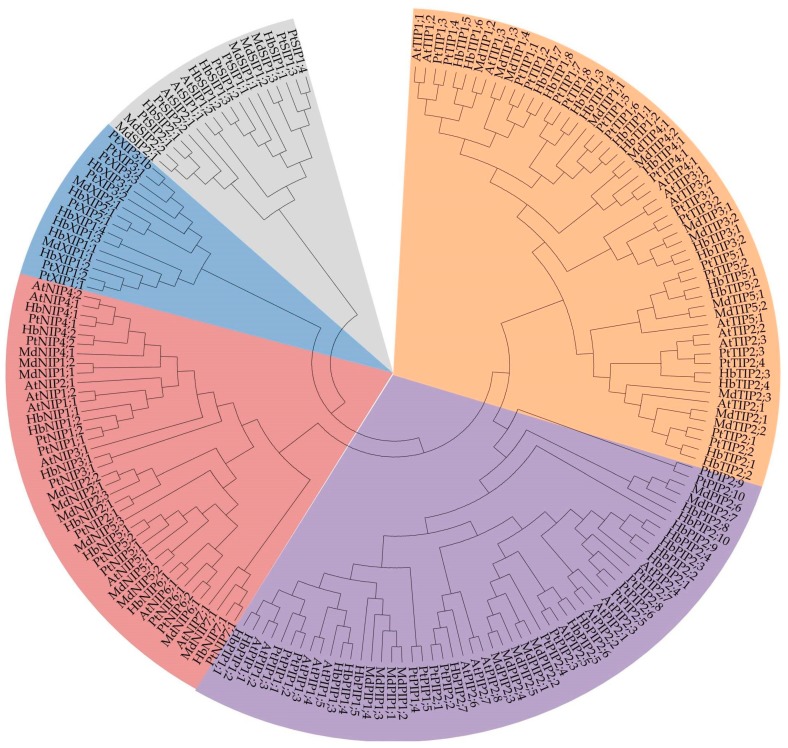
Phylogenetic tree of AQPs from *Arabidopsis thaliana*, *Populus trichocarpa*, *Hevea brasiliensis,* and *Malus domestica*. The protein sequences were aligned by ClustalX and the phylogenetic tree was constructed by the Neighbor-Joining method (1000 bootstrap replicates) in the MEGA6 software. The subgroups are marked by a colorful background (orange for TIPs, purple for PIPs, red for NIPs, blue for XIPs and gray for SIPs).

The phylogenetic relationships between the set of MdAQPs with homologous proteins encoded by *A. thaliana*, poplar, and rubber (*Hevea brasiliensis*) is displayed in Figure 1. The analysis allowed the set of apple AQPs to be each assigned membership of one of the five plant AQP subfamilies, namely the MdPIPs (eleven members), the MdTIPs (thirteen members), the MdNIPs (eleven members), the MdSIPs (five members), and the MdXIPs (two members). The MdPIP members were further classified into the two subgroups, MdPIP1 and MdPIP2, the MdSIPs into the two subgroups, MdSIP1 and MdSIP2, and the MdTIPs into the five subgroups, MdTIP1-MdTIP5. The two MdXIPs belonged into the two subgroups MdXIP1 and MdXIP2, respectively, and the MdNIPs were divided into six subgroups, MdNIP1, 2, 4, 5, 6 and 7. On the basis of sharing a level of >90% similarity at the peptide level, 14 pairs of sequences were recognized, namely MdNIP1;1/MdNIP1;2, MdNIP2;1/MdNIP2;2, MdNIP5;1/MdNIP5;2, MdTIP1;3/MdTIP1;4, MdTIP4;1/MdTIP4;2, MdTIP2;1/MdTIP2;2, MdTIP3;1/MdTIP3;2, MdTIP5;1/MdTIP5;2, MdPIP1;1/MdPIP1;2, MdPIP2;1/MdPIP2;2, MdPIP2;3/MdPIP2;4, MdPIP2;6/MdPIP2;7, MdSIP1;2/MdSIP1;3, and MdSIP2;1/MdSIP2;2.

### 2.3. Chromosomal Location and Gene Structure

**Figure 2:**
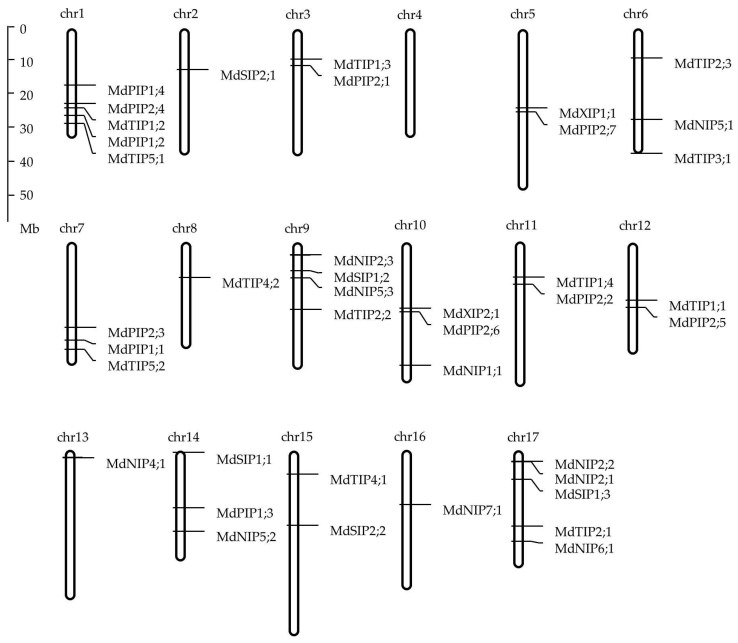
Distribution of *AQP* genes in apple chromosomes. Two genes (*MdNIP1;2* and *MdTIP3;2*) could not be localized on any chromosome. The scale is in megabases (Mb).

**Figure 3:**
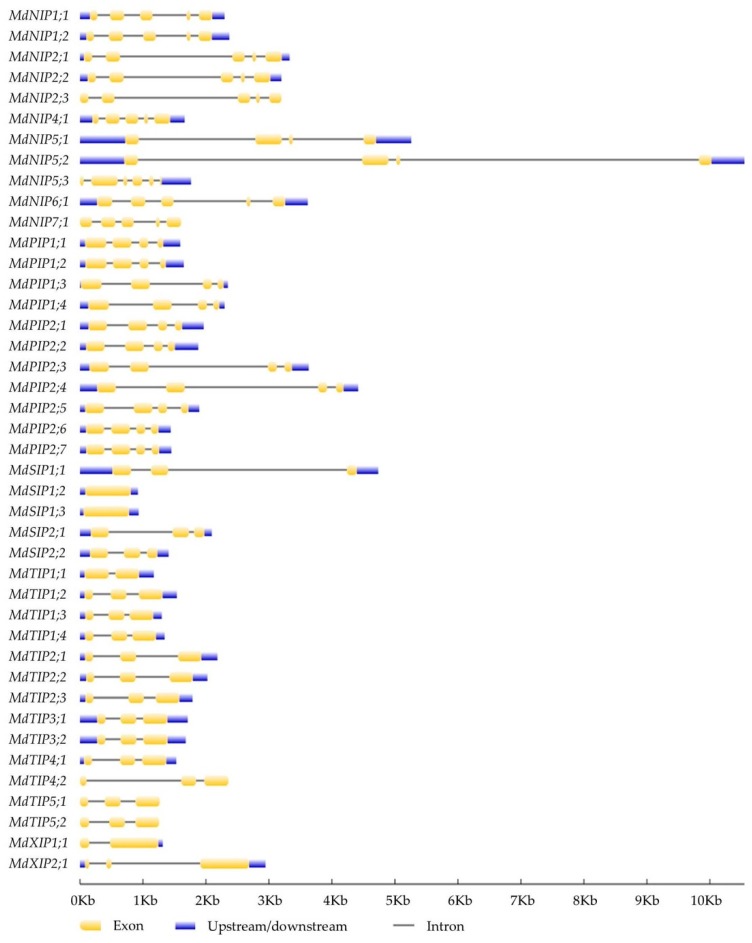
The exon-intron structure of apple *AQP* genes. Upstream/downstream region, exon, and intron are represented by blue box, yellow box, and grey line using GSDS software, respectively.

It was possible to map 40 of the 42 *MdAQPs* on 16 of the 17 apple chromosomes, but neither *MdNIP1;2* nor *MdTIP3;2* could be placed (Figure 2). The sequence of each of the eleven *MdPIPs* featured three introns; all but one of the thirteen *MdTIPs* featured two introns (the exception was *MdTIP1;1* in which only one intron was present); eight of the eleven *MdNIP* sequences were interrupted by four introns, with three introns present in both *MdNIP5;1* and *MdNIP5;2*, and five in *MdNIP5;3*; three of the five *MdSIPs* harbored two introns, while neither *MdSIP1;2* nor *MdSIP1;3* featured any introns; finally, *MdXIP1;1* had one intron while *MdXIP2;1* included two introns (Figure 3).

### 2.4. Conserved Residues in the Apple AQPs

The NPA motifs, ar/R filter, and Froger’s positions were identified via a multiple alignment between the apple AQPs and other plant AQPs (Table 2). These conserved positions were critical for the substrate selectivity of AQPs. Both NPA domains were conserved in all MdPIP and MdTIP members, but the third residue of the first NPA in MdNIP5;1, MdNIP5;2, and MdNIP5;3 was serine rather than alanine, while in MdNIP5;1, MdNIP5;2, and MdNIP6;1, the third residue of the second NPA was valine rather than alanine; in MdNIP2;3 it was glutamate and in MdNIP5;3 it was isoleucine. The MdSIPs all carried a non-conserved third residue in the first NPA, while in addition, the first residue of the second NPA in MdSIP2;2 was serine rather than asparagine. Both the first and third residues of the first NPA of MdXIP1;1 were non-conserved and the third residues of the first NPA of MdXIP2;1 was valine. The ar/R filter sequence was well conserved within each subfamily, but varied between the subfamilies. Each of the PIPs carried the conserved sequence phenylalanine-histidine-threonine-arginine. The greatest diversity for this motif was present among the NIPs, where each of the tetrapeptides tryptophan-valine-alanine-arginine, glycine-serine-glycine-arginine, alanine-isoleucine-glycine-arginine, threonine-isoleucine-alanine-arginine, and alanine-valine-glycine-arginine was represented. With respect to the Froger’s positions, there was also high conservation within each subfamily, but variability between subfamilies. While the P1 position was the only variable residue within the PIP and XIP subfamily members, both the P1 and P2 positions varied among members of TIP and SIP subfamilies, and the P1, P2, and P5 positions were were all non-conserved for NIP subfamily members.

### 2.5. The Site of Apple PIP2 Expression

**Figure 4:**
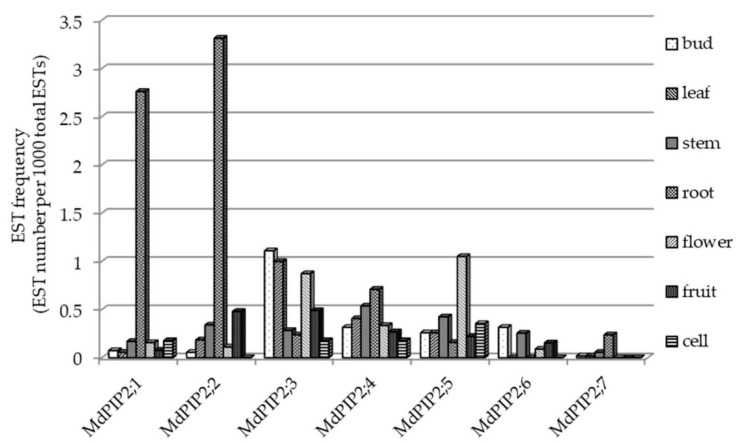
The frequency of the various *MdPIP2* ESTs present in cDNA libraries constructed from RNA extracted from buds, leaves, stems, roots, flowers, fruit and in vitro cultured cells.

**Figure 5:**
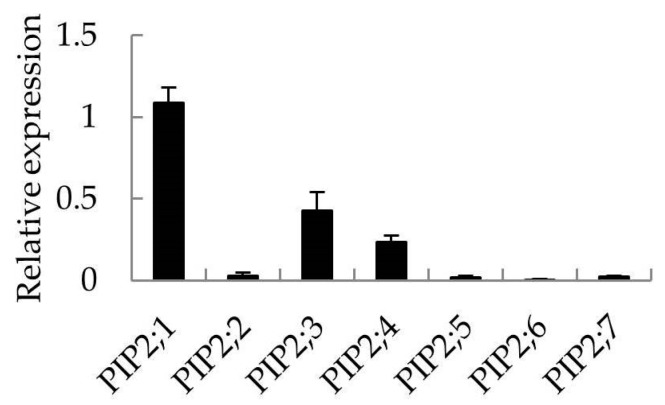
Transcriptional profiling of seven *PIP2* genes in the root of *M. hupehensis*. RNA was extracted from the roots of hydroponically-raised *M. hupehensis* seedlings which had formed 7–8 true leaves. Values show in the form mean ± SD (*n* = 3).

A search of the set of apple ESTs deposited in GenBank resulted in 685 hits for seven *MdPIP2s*, a subgroup of the *M. domestica AQP* gene family, the products of which are likely important regulators of water transport across the plasma membrane. The distribution of these hits among each *PIP2* gene and the organ are shown in Figure 4. The most well represented gene was *MdPIP2;4* (218 hits) and the least well represented was *MdPIP2;5* (seven hits). The site of transcription of these genes can be inferred from the frequency of their transcripts’ occurrence in the 76 cDNA libraries assembled from various organs. In the bud libraries, there were 116 such ESTs out of a total of 54,099 sequences; there were 104 out of 54,120 in the leaf libraries; there were 73 out of 35,380 in the stem libraries; there was 94 out of 12,679 in the root libraries; there were 117 out of 44,772 in the flower libraries; there were 176 out of 104,341 in the fruit libraries; and finally out of libraries constructed from in vitro cultured cells, there were 5 out of 5652. When the abundance of transcripts generated from seven of the *PIP2* genes was evaluated by applying a quantitative real-time PCR (qRT-PCR) assay to RNA extracted from *M. hupehensis* root tissue, *PIP2;1* appeared to be the gene most strongly transcribed (Figure 5). Since both drought and salinity stress are sensed by roots, *PIP2;1* was chosen for a detailed functional analysis. In particular, the copy present in *Malus prunifolia* was selected, as this species provides a source of drought-tolerant rootstocks [22]. 

### 2.6. The Abiotic Stress Tolerance of A. thaliana Plants Heterologously Expressing MpPIP2;1

**Figure 6:**
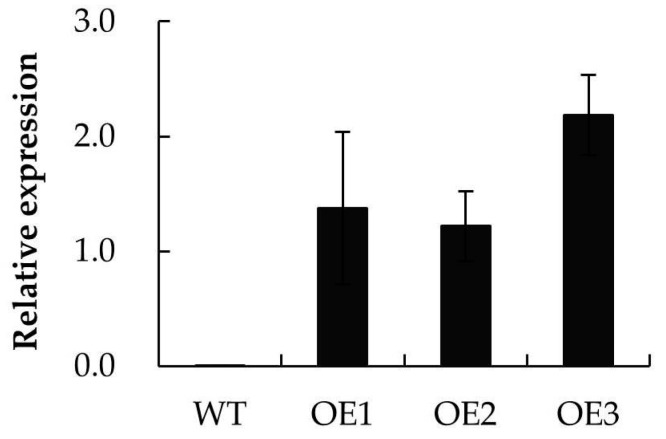
Relative expression of *MpPIP2;1* in three *A. thaliana* transgenic lines heterologously expressing *MpPIP2;1* using qRT-PCR with Col-0 as control. The value presented was the mean ± SD of three replicates.

A set of ten independent *A. thaliana* transgenics heterologously expressing *MpPIP2;1* was obtained, and three of these were randomly selected to advance to the T3 generation (Figure 6). The performance of the three transgenic lines was then compared with that of wild type (WT) Col-0 plants.

**Figure 7:**
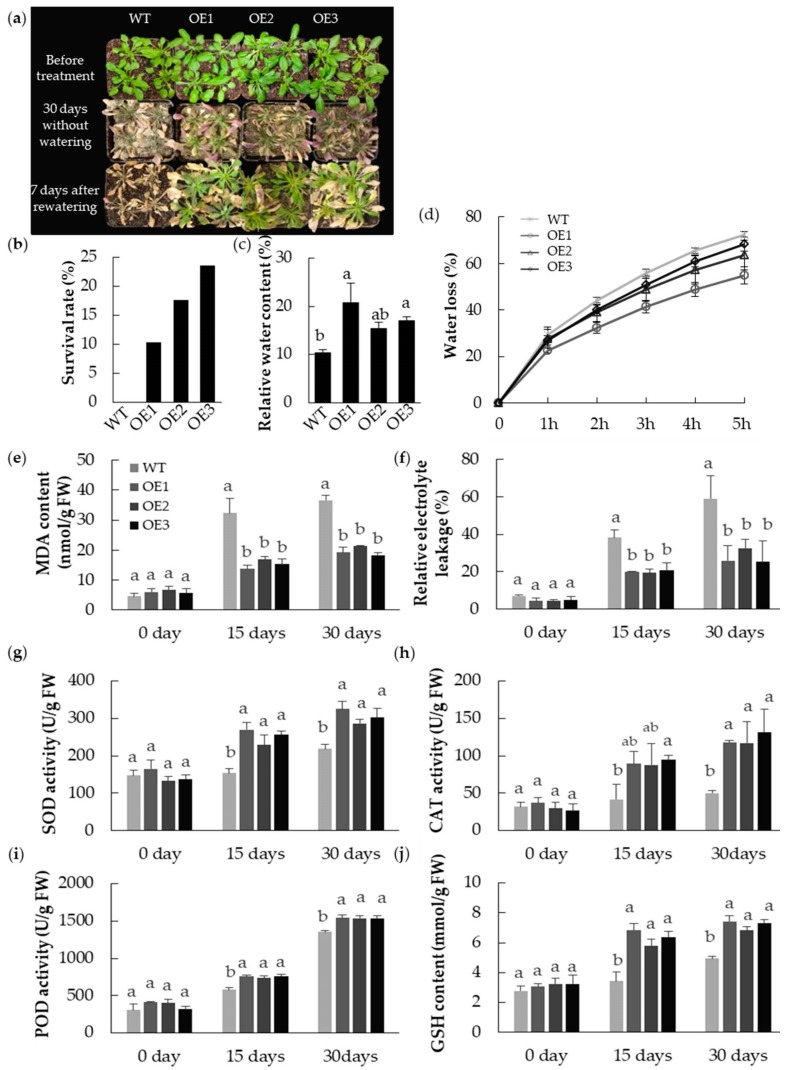
Heterologous expressing *MpPIP2;1* enhanced drought tolerance in *A. thaliana*. (**a**) Phenotypes of transgenic lines and wild type plants under drought stress; (**b**) survival rate after 30 days withholding of water and 7 days after rewatering; (**c**) relative water content after 30 days withholding of water; (**d**) water loss rate of detached leaves; (**e**) malondialdehyde (MDA) content; (**f**) relative electrolyte leakage; (**g**) superoxide dismutase (SOD) activity; (**h**) catalase (CAT) activity; (**i**) peroxidase (POD) activity; and (**j**) glutathione (GSH) content at 0, 15, and 30 days after water withheld. The value presented was the mean ± SD of three replicates, and the bar with different letter was significantly different between plants at *p* < 0.05.

The contrasting effect of drought stress on the transgenic and WT plants is illustrated in Figure 7. Under well-watered conditions, the growth of the transgenic lines was indistinguishable from that of WT plants. However, when water was withheld for 30 days, none of the WT plants remained viable as they were unable to maintain a sufficient level of leaf hydration (their relative water content fell to 10.5%); in contrast, many of transgenic plants survived, maintaining a leaf relative water content of about 20%. The post-stress survival rate of these latter plants was between 10.3% and 23.5%. A comparison of the leaf malondialdehyde (MDA) content showed that less of this stress marker accumulated in the transgenic plants than in WT plants; similarly, it was established that the relative electrolyte leakage of leaves sampled from the transgenic plants was lower than that of WT leaves. The activity of each of the enzymes superoxide dismutase (SOD), catalase (CAT), and peroxidase (POD), as well as the content of glutathione (GSH), were all greater in the transgenic plants than in the WT ones. Consistent with this result, the detached leaves of transgenic lines lost water slower than that of WT leaves. After 5 h of dehydration, the water loss rate for transgenic lines OE1, OE2, and OE3 were 17.6%, 8.8%, and 4.13% lower than that of WT, respectively.

**Figure 8:**
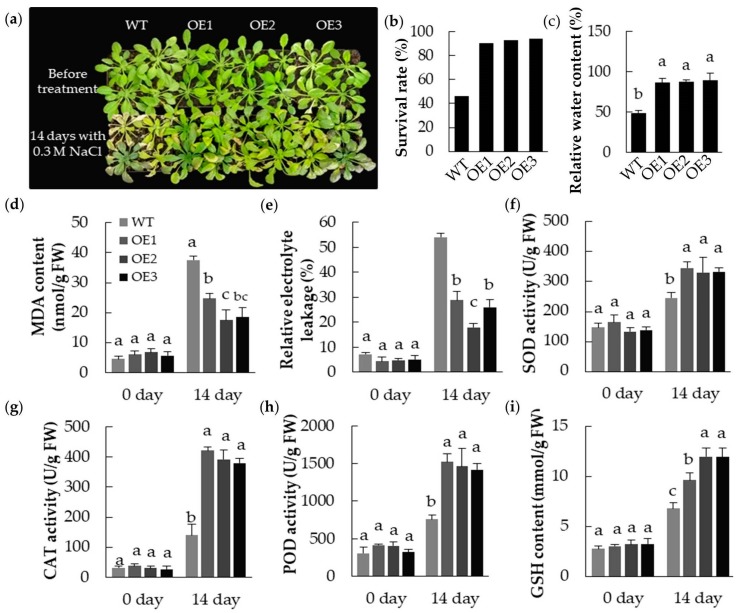
Heterologous expressing *MpPIP2;1* enhanced salt tolerance in *A. thaliana*. (**a**) Phenotypes of transgenic lines and wild type plants treated with 0.3 M NaCl for 14 days; (**b**) Survival rate and (**c**) relative water content after exposure to NaCl; (**d**) MDA content; (**e**) relative electrolyte leakage; (**f**) SOD activity; (**g**) CAT activity; (**h**) POD activity; and (**i**) GSH content at 0 and 14 days after NaCl treatment. The value presented was the mean ± SD of three replicates, and the bar with different letter was significantly different between plants at *p* < 0.05.

A series of experiments were conducted to establish whether the constitutive expression of *MpPIP2;1* in *A. thaliana* had any effect on the level of tolerance to salinity stress, as imposed by exposure to 0.3 M NaCl for 14 days (Figure 8). The transgenic plants maintained a superior leaf hydration status compared to the WT plants: Their respective relative water contents were >80% and 49%. While the survival rate of WT plants was 46.4%, that of the transgenic plants was >90%. Compared to WT leaves, those of the transgenic plants accumulated less MDA, developed a lower relative electrolyte leakage, exhibited a higher activity of SOD, POD, and CAT, and their GSH content was greater.

### 2.7. Germination and Root Elongation of MpPIP2;1 Transgenics Exposed to Either Salinity or Osmotic Stress

**Figure 9:**
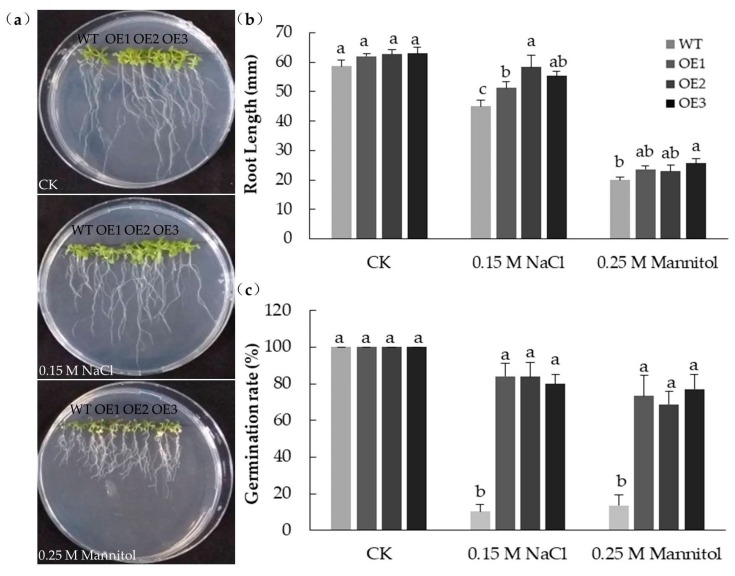
Heterologous expressing *MpPIP2;1* enhanced seeds germination and root elongation in *A. thaliana* either under salinity or osmotic stress. (**a**) The phenotype and (**b**) the statistical analyses of the root lengths of transgenic lines and wild type seedlings growing on MS medium (CK), or MS medium with 0.15 M NaCl or 0.25 M mannitol for 14 days; (**c**) germination rate of transgenic and wild type seeds on different mediums for 7 days. The value presented was the mean ± SD of three replicates, and the bar with different letter was significantly different between plants at *p* < 0.05.

An experiment was conducted to establish whether the constitutive expression of *MpPIP2;1* in *A. thaliana* had any effect on germination and/or root elongation in the presence of either salinity or osmotic stress (Figure 9). When the seed was imbibed in the absence of a stress agent (mannitol or NaCl), the rate of germination of both the WT and transgenic seed was high, and there were no significant differences between the germination rates of WT and transgenic seeds. However, in the presence of either 0.25 M mannitol or 0.15 M NaCl, the rate of germination of the WT seeds fell to just 10%, while that of the transgenic seeds remained >60%. Similarly, the ability of roots to elongate was the same for the WT and transgenic seedlings under non-stressful conditions, but the extent of its inhibition by the presence of either mannitol or NaCl differed between the transgenic and WT seedlings.

## 3. Discussion

The systematic scanning of the content of AQP-encoding genes in the apple genome reported here resulted in the identification of 42 such genes. AQPs make an important contribution to the way in which plants control their uptake of water, and hence represent a key component of their response to drought and osmotic stress [23]. Thus, gaining a full understanding of how apple plants regulate their water balance and adapt to drought and osmotic stress will likely involve revealing the function of many of this set of genes. There is already some experimental evidence which supports the participation of *AQPs* in the stress response of apple. According to Hu et al. (2003), the transcription of both *MdPIP1a* and *MdPIP1b* (here renamed as, respectively, *MdPIP1;2* and *MdPIP1;1*) are up-regulated by osmotic stress [24]. The *M. zumi* homolog of *MdPIP1;1* has been shown to be inducible by salinity (as well as by low temperature) stress [25]. Meanwhile, the constitutive expression of *MzPIP2;1* (homolog of *MdPIP2;4*) in *A. thaliana* has a positive effect on drought tolerance and a small positive one on salinity tolerance [26], and the expression of *MzPIP1;3* (homolog of *MdPIP1;3*) in tomato has been shown to enhance the plants’ drought tolerance [27].

Both moisture and nutrient stress are initially sensed by a plant’s roots. Based on its relatively high transcript abundance (inferred both indirectly from the frequency of its representation in EST libraries and directly through a qRT-PCR analysis), *PIP2;1* is the *PIP2* gene most strongly transcribed in the roots of the apple plant. This same gene has been shown, using a suppression subtractive hybridization method, to be up-regulated in response to moisture deficit [28], while two *MdPIP2;1* ESTs have been identified in cDNA libraries developed from the roots of plants from which water had been withheld for a week (LIBEST_024527). Thus the evidence points to the conclusion that the product of *PIP2;1* is an important component of the apple plant’s response to moisture stress. This evidence has been strengthened by the demonstration here that heterologously expressing the gene in *A. thaliana* had a positive effect on the plant’s tolerance of both drought and salinity.

A commonly observed plant response to abiotic stress is to accumulate reactive oxygen species (ROS), which become cytotoxic when present in excess [29]. When *A. thaliana* plants heterologously expressing *MpPIP2;1* were exposed to either drought or salinity stress, both the MDA content and the relative electrolyte leakage of their leaves were below the levels shown by WT leaves; both of these traits are correlated with ROS-mediated cellular damage [30]. The maintenance of a non-damaging level of cellular ROS content is achieved both by the activity of a number of enzymes and the synthesis of antioxidant compounds [31]. Both the activity of the enzymes SOD, CAT, and POD and the content of the antioxidant compound GSH were higher in the transgenic than in the WT *A. thaliana* plants subjected to stress. The conclusion is that the product of *MpPIP2;1* likely contributes to protecting the transgenic plants experiencing drought stress by enhancing their ability to control the accumulation of ROS. Similar conclusions have been drawn with respect to AQPs in a number of plant systems [32], so it is reasonable to propose that the product of *MpPIP2;1*, a gene which is strongly transcribed in the root of *M. prunifolia*, is an important determinant of the drought stress response when expressed in its native context.

## 4. Materials and Methods 

### 4.1. Identification of the Set of AQP Genes in the Apple Genome

Apple AQP sequences were recovered from the NCBI Protein database (www.ncbi.nlm.nih.gov/protein) by entering as a keyword search “(aquaporin OR MIP) AND Malus”. The resulting hits were confirmed as genuine AQPs by submitting them to the NCBI Conserved Domain database (www.ncbi.nlm.nih.gov/Structure/cdd/wrpsb.cgi). The sequences of identified apple AQPs were used as queries to search Malus x domestica Whole Genome v1.0 and GDDH13 Whole Genome v1.1 sequence for additional members with an E value less than 0.01. A phylogenetic analysis based on the deduced peptide sequences of AQPs encoded by *A. thaliana*, poplar, and rubber [33] was used assign the apple sequences to the five established AQP subfamilies. Multiple sequence alignments were carried out using ClustalX software [34], and an unrooted phylogenetic tree was constructed using MEGA6 software [35], applying the Neighbor-Joining method and 1000 bootstrap replicates.

### 4.2. Chromosomal Location, Gene Structure, and Protein Properties of Apple AQPs

The GDDH13 assembly was used to reveal the chromosomal location for each of the *MdAQPs* and to determine the intron/exon structure of each gene. The latter was visualized using GSDS software (bio.tools/GSDS) [36]. The pI and molecular weight of the deduced AQPs were predicted using the ExPASY program (web.expasy.org/compute_pi/). Transmembrane regions were detected using TMHMM software (www.cbs.dtu.dk/services/TMHMM/) [37], and subcellular localizations were predicted using WoLF PSOR software (wolfpsort.hgc.jp/) [38]. Sequences representing conserved domains, NPA motifs, the ar/R filter, and the Froger positions were manually identified, based on multiple sequence alignments of apple AQPs with heterologous AQPs.

### 4.3. The Sites of Apple PIP2 Expression

Apple ESTs were retrieved through a BLASTN search of the GeneBank database, using as search terms each of the *MdPIP2* transcripts in turn. RNA was extracted from the roots of hydroponically-raised *M. hupehensis* seedlings (a triploid species characterized by facultative apomixis) which had formed 7–8 true leaves [39], and was processed for a series of qRT-PCR assays targeting seven *PIP2* genes. The relevant primers were designed using Beacon Designer 8 are shown in Table 3.

### 4.4. Heterologous Expression of MpPIP2;1 in A. thaliana

A full length copy of *MpPIP2;1* cDNA (JF834203.1) was PCR-amplified from an in-house p*MD19-T-MpPIP2;1* plasmid using a primer pair listed in Table 3 [41]. This was used to generate the construct p*CAMBIA2300-35S-MpPIP2;1*, which was introduced into *A. tumefaciens* strain GV3101, and from thence into *A. thaliana* (ecotype Col-0) using the floral dip method [42]. Selection for transgenic products was carried out by culturing on a medium containing 50 mg·L^−1^ kanamycin. The abundance of *MpPIP2;1* transcript produced in transgene homozygous T3 lines was evaluated using a qRT-PCR assay based on the primer pairs listed in Table 3.

### 4.5. Stress Tolerance Analysis

For the purpose of assaying in vitro germination, surface-sterilized WT and transgenic seeds were laid on either solidified Murashige and Skoog (1962) medium (MS) [43], MS containing 0.25 M mannitol, or MS containing 0.15 M NaCl, and held for seven days under a 16 h photoperiod at 23 °C. Root elongation was assessed by culturing pre-germinated seedlings for two weeks on vertically oriented plants containing MS, MS + 0.25 M mannitol, or MS + 0.15 M NaCl. To test for both drought and salinity tolerances, seedlings were grown in pots containing equal amounts of soil after stratified and generated in soil for 3 weeks. The water loss rate was measured by weighing the detached leaves at different time points. The plants were then either subjected to drought by the withholding of water for 30 days, or salinity stress by irrigation with 0.3 M NaCl solution [44,45]. The assays for relative leaf water content, leaf electrolyte leakage, and MDA concentration have been described elsewhere. To assay for SOD, CAT, and POD activity and GSH content, leaves was homogenized in phosphate buffer (pH 7.5) and the resulting supernatants recovered after centrifugation were tested using commercially available kits purchased from Nanjing Jiancheng Bioengineering Institute (Nanjing, China). The SOD Assay Kit was based on hydroxylamine method, the CAT Assay Kit was based on the method of ammonium molybdate, the POD Assay Kit was based on the hydrogen peroxide oxidation reaction, and the GSH assay kit is based on reduced glutathione reacting with 5,5′-dithiobis-2-nitrobenoic acid.

### 4.6. Statistical Analysis

Data were statistically analyzed using routines implemented in SPSS v17.0. Means were compared using Tukey’s test, applying a 0.05 probability threshold to declare significance.

## 5. Conclusions

The present experiment has identified that the apple genome harbors 42 genes encoding putative AQPs. It is necessary for the future to comprehensively understand the process of water and small solutes flues through the membrane and related molecular-regulating mechanisms in the apple tree. In *A. thaliana,* plants were engineered to heterologously express one of these genes (*MpPIP2;1*), a gene which is strongly transcribed in the root of *M. prunifolia*. The transgenic plants exhibited an improved level of tolerance to both drought and salinity stress, which implies that in the apple, the product of *PIP2;1* is an important component of the plant’s response to moisture stress. This latter finding suggests a strategy for enhancing the stress tolerance of the apple using a molecular approach.

## Figures and Tables

**Table 1:** The information of identified apple AQPs.

Name	GeneBank accession no.	Gene ID	Size(aa)	MW(Da)	pI	TMD	Loc
MdNIP1;1	XP_008383590.1		271	28742.49	9.03	6	Plas
MdNIP1;2	XP_028954386.1		271	28743.38	8.99	6	Plas
MdNIP2;1	XP_008341577.1		290	30456.02	6.70	6	Plas
MdNIP2;2	XP_008341755.2		290	30513.07	6.70	6	Plas
MdNIP2;3		MD09G1051500 **^1^**	260	27306.65	6.65	6	Plas
MdNIP4;1		MD13G1025400 **^1^**	278	29945.16	8.97	7	Plas
MdNIP5;1	XP_008356814.1		298	30960.84	8.94	5	Vacu
MdNIP5;2	XP_008360732.1		298	31052.15	8.85	6	Vacu
MdNIP5;3	XP_008348330.2		266	27575.92	9.23	5	Plas
MdNIP6;1	XP_008343681.1		306	31641.74	8.90	6	Vacu
MdNIP7;1	XP_028952006.1		300	31457.78	8.05	6	Plas
MdPIP1;1	NP_001280914.1		289	30875.87	9.32	5	Plas
MdPIP1;2	NP_001280922.1		289	30849.79	9.30	6	Plas
MdPIP1;3	NP_001315794.1		286	30759.57	9.08	6	Plas
MdPIP1;4	NP_001280950.1		286	30660.58	9.15	6	Plas
MdPIP2;1	XP_008363507.1		281	30166.00	7.65	6	Plas
MdPIP2;2	XP_008385311.2		281	30179.06	8.25	6	Plas
MdPIP2;3	XP_008365039.2		287	30461.39	8.95	6	Plas
MdPIP2;4	XP_008367680.2		283	30050.96	8.68	6	Plas
MdPIP2;5	XP_008387595.1		285	30156.86	6.89	6	Plas
MdPIP2;6	XP_008382110.2		281	29856.60	9.08	6	Plas
MdPIP2;7	XP_008377729.1		281	29924.69	9.22	6	Plas
MdSIP1;1	XP_008357207.2		242	25827.57	9.55	6	Vacu
MdSIP1;2	XP_008348137.2		240	25347.21	9.96	5	Vacu/Plas
MdSIP1;3	XP_008342016.1		240	25427.44	9.97	5	Vacu
MdSIP2;1	XP_008354672.2		236	25496.16	9.17	5	Vacu
MdSIP2;2	XP_008338069.3		236	25364.97	9.48	6	Chlo
MdTIP1;1	XP_008387528.2		252	26029.00	5.18	6	Plas
MdTIP1;2	XP_008343557.2		252	26029.25	5.62	6	Plas
MdTIP1;3	XP_008357781.1		252	25944.08	4.96	6	Plas
MdTIP1;4	XP_008366336.1		252	25922.12	4.96	6	Plas
MdTIP2;1	XP_008342659.1		248	25234.34	5.76	7	Plas
MdTIP2;2	XP_008380900.2		248	25308.41	5.62	7	Plas
MdTIP2;3	XP_008373770.1		248	25109.15	4.86	6	Vacu
MdTIP3;1	XP_008351935.2		256	26996.43	7.10	6	Plas
MdTIP3;2		MDP0000868372 **^2^**	255	26710.01	7.06	5	Plas
MdTIP4;1	XP_008393878.2		248	25864.24	5.51	7	Plas
MdTIP4;2		MD08G1115300 **^1^**	239	25098.22	4.98	7	Plas
MdTIP5;1	XP_008354229.2		254	26270.51	7.74	6	E.R.
MdTIP5;2	XP_008376403.3		256	26196.42	6.24	6	Chlo/plas
MdXIP1;1	XP_017182198.2		304	32165.86	5.96	6	Plas
MdXIP2;1^2^	XP_028965550.1		309	33249.21	8.21	7	Plas

^1^ Gene IDs with were based on Malus × domestica GDDH13 Whole Genome v1.1. ^2^ Gene IDs were based on Malus × domestica Whole Genome v1.0. (AQPs, aquaporins; MW, molecular weight; pI, isoelectric point; TMD, transmembrane domain; Loc, subcellular localization; Chlo, chloroplast; Plas, plasma membrane; Vacu, vacuolar membrane; E. R., endoplasmic reticulum).

**Table 2:** NPA motifs, ar/R filter, and Froger’s positons of apple AQPs.

Name	NPA motifs		Ar/R filter				Froger’s Positions				
	LB	LE	H2	H5	LE1	LE2	P1	P2	P3	P4	P5
MdNIP1;1	NPA	NPA	W	V	A	R	F	S	A	Y	I
MdNIP1;2	NPA	NPA	W	V	A	R	F	S	A	Y	I
MdNIP2;1	NPA	NPA	G	S	G	R	L	T	A	Y	V
MdNIP2;2	NPA	NPA	G	S	G	R	L	T	A	Y	V
MdNIP2;3	NPA	NPE	G	S	-	R	L	T	A	Y	V
MdNIP4;1	NPA	NPA	W	V	A	R	L	S	A	Y	F
MdNIP5;1	NPS	NPV	A	I	G	R	F	T	A	Y	L
MdNIP5;2	NPS	NPV	A	I	G	R	L	T	A	Y	L
MdNIP5;3	NPS	NPI	A	I	G	R	F	T	A	Y	L
MdNIP6;1	NPA	NPV	T	I	A	R	F	T	A	Y	L
MdNIP7;1	NPA	NPA	A	V	G	R	Y	S	A	Y	I
MdPIP1;1	NPA	NPA	F	H	T	R	E	S	A	F	W
MdPIP1;2	NPA	NPA	F	H	T	R	E	S	A	F	W
MdPIP1;3	NPA	NPA	F	H	T	R	Q	S	A	F	W
MdPIP1;4	NPA	NPA	F	H	T	R	Q	S	A	F	W
MdPIP2;1	NPA	NPA	F	H	T	R	Q	S	A	F	W
MdPIP2;2	NPA	NPA	F	H	T	R	Q	S	A	F	W
MdPIP2;3	NPA	NPA	F	H	T	R	Q	S	A	F	W
MdPIP2;4	NPA	NPA	F	H	T	R	Q	S	A	F	W
MdPIP2;5	NPA	NPA	F	H	T	R	Q	S	A	F	W
MdPIP2;6	NPA	NPA	F	H	T	R	Q	S	A	F	W
MdPIP2;7	NPA	NPA	F	H	T	R	Q	S	A	F	W
MdSIP1;1	NPT	NPA	V	L	P	N	M	A	A	Y	W
MdSIP1;2	NPS	NPA	S	L	P	N	M	A	A	Y	W
MdSIP1;3	NPS	NPA	S	L	P	N	M	A	A	Y	W
MdSIP2;1	NPL	NPA	S	L	G	S	F	V	A	Y	W
MdSIP2;2	NPL	SPA	S	L	G	S	F	V	A	Y	W
MdTIP1;1	NPA	NPA	H	I	A	V	T	S	A	Y	W
MdTIP1;2	NPA	NPA	H	I	A	V	T	S	A	Y	W
MdTIP1;3	NPA	NPA	H	I	A	V	T	S	A	Y	W
MdTIP1;4	NPA	NPA	H	I	A	V	T	S	A	Y	W
MdTIP2;1	NPA	NPA	H	I	G	R	T	S	A	Y	W
MdTIP2;2	NPA	NPA	H	I	G	R	T	S	A	Y	W
MdTIP2;3	NPA	NPA	H	I	G	R	T	S	A	Y	W
MdTIP3;1	NPA	NPA	H	I	A	R	T	A	A	Y	W
MdTIP3;2	NPA	NPA	H	I	A	R	T	A	A	Y	W
MdTIP4;1	NPA	NPA	H	I	A	R	S	S	A	Y	W
MdTIP4;2	NPA	NPA	H	I	A	R	S	S	A	Y	W
MdTIP5;1	NPA	NPA	N	V	G	C	T	A	A	Y	W
MdTIP5;2	NPA	NPA	N	V	G	C	I	A	A	Y	W
MdXIP1;1	SPV	NPA	V	V	V	R	M	C	A	F	W
MdXIP2;1	NPV	NPA	I	T	V	R	V	C	A	F	W

NPA, Asparagine-Proline-Alanine; Ar/R, aromatic/arginine; LE, loop E; LB, Loop B; H2, transmembrane helix 2; H5, transmembrane helix 5.

**Table 3:** Primers used in this study.

Gene Name ^1^	Sequence (5′-3′)	Use	Reference
*MpPIP2;1*	F: ATGGCAAAAGATATTGAGGG	Gene cloning	This study
	R: TTAAGCATTGCTCCTGAAAG		
	F: CTTGGCTCGCAAGGTTTCAC	qRT-PCR for *MpPIP2;1* in transgenic *Arabidopsis*	This study
	R: GTAGCCATCAGCCAACTCGT		
*AtACTIN*	F: CCTTCTACCACCAATACATTC	qRT-PCR for reference gene	[40]
	R: TGTTCCATTGTCGCATAC		
*MhPIP2;1*	F:CCTTCTACCACCAATACATTC	qRT-PCR for *MhPIP2;1*	This study
	R:TGATTATCTACAATTCCATAGCC		
*MhPIP2;2*	F:GCGGTGGAACTGTAGATA	qRT-PCR for *MhPIP2;2*	This study
	R:GCTTTCTCTGGCATCAAT		
*MhPIP2;3*	F:CAAGAGGAGTGCTAGAGAC	qRT-PCR for *MhPIP2;3*	This study
	R:GCCAAGTGGACAATGAAC		
*MhPIP2;4*	F:CTTGGACCTGCTGTTATCT	qRT-PCR for *MhPIP2;4*	This study
	R:AATTGCTGCTCCGATGAA		
*MhPIP2;5*	F:TGGATTATTCTGGAAGCAT	qRT-PCR for *MhPIP2;5*	This study
	R:GCAACATTAAGGCACATT		
*MhTIP2;6*	F:GCAACCCGACCCACTAAA	qRT-PCR for *MhPIP2;6*	This study
	R:ACAACACTCTCAATACACACTACA		
*MhTIP2;7*	F:CAGCAACCCAACCAACTAAA	qRT-PCR for *MhPIP2;7*	This study
	R:ATCATCATCCATCCTCTCTCAAT		
*MhACTIN*	F:TTCGTTTTCGTTTTCGTTTT	qRT-PCR for reference gene	This study
	R:TGTTCCATTGTCGCATAC		

^1^ Primers were designed to target the seven *MhPIP2* based on the sequences of their homologs in *M. domestica*.

## Abbreviations

AQP	aquaporins
NPA	asparagine-proline-alanine
ar/R	aromatic/arginine
NIP	Nod26-like intrinsic proteins
PIP	plasma membrane intrinsic proteins
SIP	small and basic intrinsic proteins
TIP	tonoplast intrinsic proteins
XIP	uncharacterized intrinsic proteins
MDA	malondialdehyde
SOD	superoxide dismutase
CAT	catalase
POD	peroxidase
GSH	glutathione
